# Impact of Baseline Characteristics on the Effectiveness of Disorder-Specific Cognitive Behavioral Analysis System of Psychotherapy (CBASP) and Supportive Psychotherapy in Outpatient Treatment for Persistent Depressive Disorder

**DOI:** 10.3389/fpsyt.2020.607300

**Published:** 2020-12-21

**Authors:** Ilinca Serbanescu, Matthias Backenstrass, Sarah Drost, Bernd Weber, Henrik Walter, Jan Philipp Klein, Ingo Zobel, Martin Hautzinger, Ramona Meister, Martin Härter, Elisabeth Schramm, Dieter Schoepf

**Affiliations:** ^1^Department of Clinical Psychology and Psychotherapy, University of Heidelberg, Heidelberg, Germany; ^2^Institute of Clinical Psychology, Hospital Stuttgart, Stuttgart, Germany; ^3^Department of Psychiatry and Psychotherapy, Cognitive Behavioral Analysis System of Psychotherapy Center of Competence, University of Bonn, Bonn, Germany; ^4^Institute of Experimental Epileptology and Cognition Research, University of Bonn, Bonn, Germany; ^5^Center for Economics and Neuroscience, University of Bonn, Bonn, Germany; ^6^Department of Psychiatry and Psychotherapy, Charité-University Medicine Berlin, Berlin, Germany; ^7^Department of Psychiatry and Psychotherapy, Lübeck University, Lübeck, Germany; ^8^Psychology School at the Fresenius University of Applied Sciences Berlin, Berlin, Germany; ^9^Department of Psychology, Clinical Psychology and Psychotherapy, Eberhard Karls University Tübingen, Tübingen, Germany; ^10^Department of Medical Psychology, University Medical Center Hamburg-Eppendorf, Hamburg-Eppendorf, Germany; ^11^Department of Psychiatry and Psychotherapy, Faculty of Medicine, Medical Center-University of Freiburg, University of Freiburg, Freiburg, Germany; ^12^Department of Psychiatry and Psychotherapy, Vitos Weil-Lahn, Weilmünster, Germany

**Keywords:** persistent depressive disorder, CBASP, supportive psychotherapy, moderator analysis, predictor analysis, childhood trauma, personalized medicine

## Abstract

**Importance:** In the treatment of persistent depressive disorder (PDD), disorder-specific Cognitive Behavioral Analysis System of Psychotherapy (CBASP) has been shown to be superior to Supportive Psychotherapy (SP) in outpatients. It remains to clear which subgroups of patients benefit equally and differentially from both psychotherapies.

**Objective:** To identify those patient-level baseline characteristics that predict a comparable treatment effectiveness of CBASP and SP and those that moderate the differential effectiveness of CBASP compared to SP.

**Design, setting and participants:** In this analysis of a 48-week multicenter randomized clinical trial comparing CBASP to SP in adult antidepressant-free outpatients with early-onset PDD, we evaluated baseline variables from the following domains as potential predictors and moderators of treatment effectiveness: socio-demography, clinical status, psychosocial and global functioning, life quality, interpersonal problems, childhood trauma, treatment history, preference for psychotherapy, and treatment expectancy.

**Interventions:** A 48-week treatment program with 32 sessions of either CBASP or SP.

**Main outcomes and measures:** Depression severity measured by the 24-item Hamilton Rating Scale for Depression (HRSD-24) at week 48.

**Results:** From *N* = 268 randomized outpatients, *N* = 209 completed the 48-week treatment program. CBASP completers had significantly lower post-treatment HRSD-24 scores than SP completers (mean_CBASP_=13.96, sd_CBASP_= 9.56; mean_SP_= 16.69, sd_SP_= 9.87; *p* = 0.04). A poor response to both therapies was predicted by higher baseline levels of clinician-rated depression, elevated suicidality, comorbid anxiety, lower social functioning, higher social inhibition, moderate-to-severe early emotional or sexual abuse, no preference for psychotherapy, and the history of at least one previous inpatient treatment. Moderator analyses revealed that patients with higher baseline levels of self-rated depression, comorbidity of at least one Axis-I disorder, self-reported moderate-to-severe early emotional or physical neglect, or at least one previous antidepressant treatment, had a significantly lower post-treatment depression severity with CBASP compared to SP (all *p* < 0.05).

**Conclusions and relevance:** A complex multifactorial interaction between severe symptoms of depression, suicidality, and traumatic childhood experiences characterized by abuse, social inhibition, and anxiety may represent the basis of non-response to psychotherapy in patients with early onset PDD. Specific psychotherapy with CBASP might, however, be more effective and recommendable for a variety of particularly burdened patients compared to SP.

## Introduction

Over 20% of the patients with major depressive disorder (MDD) develop a chronic course lasting two years or longer (1), called Persistent Depressive Disorder (PDD) (2, 3). Compared to single major depressive episodes, PDD is characterized by a longer illness duration with a more complicated treatment course, lower quality of life, concurrent generalized anxiety disorder, more frequent suicide attempts, comorbid psychiatric and personality disorders, dysfunctional interpersonal behavior and more complicated treatment courses (1, 4, 5). More than two-thirds of all patients with PDD report an early illness onset (before age 21) often associated with severe experiences of childhood maltreatment characterized by emotional, physical, and sexual abuse or by deprivation in form of emotional or physical neglect (1, 4, 6, 7). Importantly, a large majority of patients with PDD experience side effects, relapses or resistances in the treatment with antidepressant medication (1, 7, 8) and report to prefer psychological over pharmacological treatment (9). Thereby, psychotherapy is an indispensable tool in the treatment of PDD.

So far, the Cognitive Behavioral Analysis System of Psychotherapy (CBASP) (10) is the only psychotherapy-model especially designed to address the specific needs of patients with early-onset PDD. Its principle lies on treating early trauma related dysfunctionalities by focusing on the patient's interpersonal problems through systematic social problem solving and discriminative interpersonal learning (10, 11). Its effectiveness has been evidenced in a number of clinical trials that compared CBASP to other psychotherapies (7), antidepressant medication (12, 13), or to combined treatments (8, 12). The European Psychiatric Association has recommended CBASP as the first-line psychotherapy for PDD, which is largely justified by its superiority over alternative, non-specific psychotherapies (5).

Nevertheless, little progress has been achieved in understanding which PDD subpopulations may or may not profit from psychotherapy in general and which benefit from CBASP in particular, leaving the questions for whom and when exactly CBASP should be recommended largely unanswered (5, 14).

This is particularly problematic, as PDD is a heterogeneous disorder, and different PDD subpopulations may benefit to varying degrees from CBASP (15). Gaining evidence is crucial not only for further explaining its general effectiveness, but also for detecting specific subpopulations for which CBASP can be recommended as first-choice psychotherapy.

One possibility to examine its disorder-specific effectiveness is by comparing it to alternative forms of psychotherapy. In a multicenter randomized clinical trial, Schramm and colleagues (7) evaluated the effectiveness of CBASP by comparing it with non-specific supportive psychotherapy (SP) in *N* = 268 antidepressant-free, adult outpatients with early-onset PDD (ClinicalTrials.gov identifier NCT00970437). Overall, CBASP was found to be more effective and acceptable than SP. Patients treated with CBASP showed small, but significant advantages in most primary and secondary outcomes, as well as in response and remission rates.

So far, a number of secondary analyses of this trial have been performed in order to analyze if CBASP outperformed SP for patients with early trauma (16), comorbid personality disorders (17), comorbid anxiety disorders (18), as well as various baseline characteristics combined to one single moderator (19). With regard to early trauma, only those patients reporting early severe-to-moderate emotional abuse seemed to benefit significantly more from CBASP than from SP at week 20 (16). The presence of comorbid personality disorders was neither a predictor nor a moderator of depression severity at week 20 (17). However, the CBASP was significantly more effective than SP in patients with comorbid anxiety disorders compared to those without anxiety disorders in terms of both depression severity and interpersonal problems as outcomes (18). In a more recent secondary analysis (19), the data of this trial was analyzed with a modern moderator approach combined with two machine learning algorithms. An optimal composite moderator (M^*^) was developed as a weighted combination of 13 preselected baseline variables and used for identifying and characterizing subgroups for which CABSP was more beneficial to SP and vice versa, focusing on the change in depression severity from baseline to week 48. Of the analyzed sample of patients, 58.65% experienced a better treatment outcome with CBASP, while 41.35% showed a better outcome with SP. In terms of baseline characteristics, patients responding more favorably to CBASP were more severely depressed, had more often a comorbid Axis-I disorder, were more often previously hospitalized, and were more likely affected by moderate-to-severe early emotional or physical neglect. In contrast, patients responding more favorably to SP had a higher pre-treatment global and social functioning level, a higher quality of life, and more often a recurrent MDD without complete remission between the episodes.

An important outstanding question which remains to be clarified is which subgroups of patients respond to both therapies. The main goal of this analysis will therefore be to identify *predictors*, i.e. baseline variables which predict treatment success regardless of treatment assignment. Discovering predictors is especially helpful for understanding which factors contribute to non-response to psychotherapy and consequently to the persistent course in chronically depressed patients. In contrast to the common practice of limiting analyses to a few characteristics and in order to gain a complex understanding, we investigated a large span of baseline characteristics including socio-demography, clinical status, psychosocial and global functioning, quality of life, interpersonal problems, childhood trauma, treatment history, preference for psychotherapy, and treatment expectancy.

Baseline characteristics which have been previously associated with a better treatment response for psychotherapy in patients with PDD and thus plausible to have contributed to a greater alleviation of depression severity in both arms are: lower baseline levels of depression and anxiety (20), having a preference for psychotherapy at the baseline (21, 22), as well as a positive treatment expectancy at baseline (23). We therefore expected an equally high effectiveness of both therapies in patients characterized by these features at baseline.

In addition, the present analysis will also examine the same baseline variables as moderators of differential treatment effectiveness of CBASP vs. SP at week 48. This will be done for statistical reasons (for determining if a variable is a predictor, one has to examine its interaction effect with the group variable), as well as for reasons of comparability with the previous moderator analysis (19) which was based on a more modern approach. Statistical models such as the one applied in the previous analysis (19), which are based on integrating several multi-domain baseline variables into one moderator to identify subpopulations with different treatment responses, are particularly useful for the prediction of treatment response in samples which are sufficiently statistically powered, and can be further validated as a prediction algorithm in new clinical populations. In comparison, the more classical approach of selecting and testing one baseline variable as predictor and moderator per model, which will be used in the analysis presented here, provides evidence about the individual impact of single baseline characteristics on the treatment outcome. These findings can further be used for selecting those clinical subpopulations which seem to respond particularly poorly to one or both therapies for testing new treatments or combination of treatments, which can be especially developed to target their needs (for instance, patients with childhood trauma, or comorbid anxiety). As for moderators, in view of its emphasis in treating cognitive-behavioral consequences of childhood trauma and previous moderator findings (19), we expected CBASP to outperform SP in reducing depression severity in patients marked by an elevated baseline depression severity, at least one comorbid Axis-I disorder, experiences of early emotional or physical neglect, lower quality of life, a longer illness duration, and those which were separated, divorced or widowed. Conversely, we expected to replicate those moderators of a higher effectiveness of SP vs. CBASP, which were: a recurrent MDD without remission between the episodes, having at least one comorbid Axis-II disorder, and a higher social and global functioning at baseline. Although these variables were not defined as moderators by testing for statistical significance in the previous approach (19), but by their moderator effect size, we expect many of them to significantly interact with the group variable in the present analysis.

## Methods

### Participants

As described in (7), eligible outpatients were fluent in the German language, 18–65 years old and met *DSM-IV* criteria for a current episode of chronic major depressive disorder (MDD) with a total duration of at least two years, MDD superimposed on a preexisting dysthymic disorder (“double depression”), or a recurrent MDD with incomplete remission between two major depressive episodes (MDEs) with a current MDD and a total duration of at least 2 years. In addition, an early illness onset (i.e. before the age of 21) and a score of at least 20 on the 24-item version of the Hamilton Rating Scale for Depression (HRSD-24) (24) at screening as well as a 2-week medication-free period at baseline were required for inclusion. Patients were excluded from study participation if they had an acute risk for suicide and/or the need for hospitalization; a primary diagnosis of another Axis I disorder; a diagnosis of antisocial, schizotypal, or borderline personality disorder; a serious medical condition; severe cognitive impairment; a history of psychotic symptoms, bipolar or organic brain disorder; an absence of a response to a previous adequate trial with CBASP and/or SP; or an ongoing psychotherapy or antidepressant medication. Intake of antidepressant medication during the trial was forbidden.

From the *N* = 622 patients assessed for eligibility, *N* = 268 met inclusion criteria and were randomized to receive CBASP (*N* = 137) or SP (*N* = 131). For further details on the inclusion process, refer to the chart flow of the main publication (7). The study was approved by the Ethics Committee of the following participating institutions: University of Freiburg, University of Bonn, University of Heidelberg, University of Tübingen, University Medical Center Hamburg-Eppendorf, University of Marburg, and University of Lübeck. Written informed consent was obtained from all participants.

### Interventions

During the entire duration of the study, both CBASP and SP were each applied following a standardized treatment manual: The CBASP was applied based on a manual developed by James P McCullough (10), while SP was applied by a revised manual developed by John C Markowitz, which was translated into German by the trial coordinators. Eligible participants were allocated to one of the intervention groups by a 1:1 treatment ratio drawing on a computer-generated block randomization sequence with randomly varying block size, stratified for trial site.

The CBASP is a highly structured psychotherapy especially developed for treating patients with chronic depression. It builds on techniques such as situation analysis, interpersonal discrimination exercises, and behavioral skill training/rehearsal (25). It was designed to address the typical preoperational cognitive-emotive functioning of patients with chronic depression by demonstrating to patients that their behavior has (negative) consequences on their environment, leading to interpersonal difficulties. Predominantly relying on the administration of negative reinforcement, CBASP supports the patient in the process of recognizing and understanding the consequences of one's behavior on their environment, which, in turn, leads to a modification of one's behavior and, consequently, to an alleviation of chronic depression. In comparison to the widely used Cognitive Behavioral Therapy (CBT), the CBASP focuses primarily on the person's behavior and interaction with its environment, and not on the pure cognitive content, which is the case for CBT (26). There is strong evidence supporting the effectiveness of CBASP with or without antidepressant medication in early-onset chronic depression: For instance, one large study (27) demonstrated that CBASP was particularly effective for the subgroup of chronically depressed patients marked by early trauma when compared to Nefazodone as antidepressant medication (remission rates: 33% with Nefazodone, 48% with CBASP, and 54% with a combination of both). Moreover, in a trial (11) conducted in *N* = 30 chronically depressed outpatients with early onset, statistically significant differences were found between CBASP and Interpersonal Therapy (IPT) regarding remission rates (57% in CBASP vs. 20% in IPT) and the decrease of self-rated depressive symptoms in favor of CBASP.

In contrast, SP is a disorder non-specific, non-confrontational psychotherapy. The supportive therapist builds an emotional connection to the patient, follows his affect, encourages catharsis, inspires hopes, and emphasizes patient's strengths (28). The main effect of this approach is the enforcement of the patient's awareness of its self-efficacy in changing its own circumstances. In a 16-week study conducted in *N* = 94 patients with dysthymia, which is a milder form of PDD, SP equaled IPT in treatment effect (29).

In an earlier trial (8), CBASP did not prove to be superior to SP when applied as a short-term (12 sessions) augmentation strategy in chronically depressed patients who showed partial or non-response to a pharmacotherapy algorithm. The present study comparing CBASP to SP was designed in order to meet the need for more and larger trials in patients with early-onset PDD, controlling for medication, and including CBASP as a disorder-specific intervention with a more intensive (larger number of sessions) and a longer course of treatment to unfold beneficial and lasting effects in PDD. In this trial, during the acute treatment phase, patients received bi-weekly sessions of CBASP or SP in the first four weeks and weekly sessions for the next 16 weeks. For the following 28 weeks, eight further continuation sessions were delivered, resuming in a total of 32 sessions extended over 48 weeks.

Both the CBASP (*N* = 42 study therapists) and SP (*N* = 39 study therapists) sessions were conducted by psychotherapists or psychiatrists with experience in the treatment of depression (mean of 5.45 years for CBASP; mean of 4.00 years for SP). Age, gender, and experience of the therapists were similar in both study conditions. All study therapists had completed a 3-year, post-graduate psychotherapy training program or were in an advanced stage of their training. In addition, both groups of study therapists were trained in CBASP or SP during a 2-day training workshop. Before treatment start, study therapists' mastery of CBASP or SP methods was assessed by specific rating scales during two videotaped pilot cases (7).

The fidelity of the therapists to the therapy manuals was measured by adherence scales including standardized scales for disciplined personal involvement and situation analysis for the CBASP. Therapy sessions of both interventions were videotaped and reviewed by site supervisors regularly on a random basis to assess psychotherapists' fidelity to the treatment procedures. In addition, an independent team of trained expert raters randomly evaluated one video-taped session of each therapy. The evaluations revealed that of *N* = 244 evaluable sessions (*N* = 123 in CBASP and *N* = 121 in SP), *N* = 227 (93.0%; with *N* = 112 in CBASP and *N* = 115 in SP) met criteria for fidelity.

In order to ensure compliance with ethical principles and the study protocol, as well as to check data quality and accuracy, monthly telephone conferences, semi-annual Data and Safety Monitoring Board conferences, and annual monitoring visits at trial sites were conducted by the Principal Investigator in cooperation with all trial site coordinators (7).

### Measurements

All ratings were performed by trained and experienced raters. Raters were furthermore blinded to patients' treatment allocation in order to avoid their possible subjective influence on the rating. For ensuring the blinding of raters, they were separately located from the therapists. In addition, patients were instructed not to mention any information that could reveal their intervention to their rater. Furthermore, back-up raters were provided in case of unintentional unblinding (7).

The HRSD-24 was used to screen for participants' eligibility before randomization (approx. two weeks before treatment start), as a main outcome after 12 and 20 weeks of acute treatment, as well as at the end of the extended treatment phase, which was 48 weeks after randomization. The interrater reliability for the HRSD-24 scores was measured based on data from 21 evaluators who rated nine audio- or video-taped interviews (intra-class correlation coefficient, 0.973; 95% CI, 0.889–0.999). Further baseline variables which were rated and subject to the present secondary analysis are described in the following section.

### Analyzed Baseline Characteristics

In the present secondary analysis of the trail by Schramm et al. (7), we tested the following baseline characteristics as potential predictors and moderators of depression severity measured by the HRSD-24 at week 48.

#### Socio-Demographic Characteristics

Gender (female/ male), age at the time point of randomization (years), marital status (single/ married or cohabiting/ separated, divorced or widowed), high educational level (corresponding to at least 12 years of education in the German school system with the possibility of university studies), employment status (employed/unemployed), working hours per week, and the presence of at least one physical illness (yes/no).

#### Clinical Characteristics

Illness subtype (chronic MDD, “double depression,” or recurrent MDD with incomplete remission between episodes), age at illness onset (years), illness duration (years), baseline severity of depression by patients' self-rating using the Inventory of Depressive Symptomatology (IDS-SR) (30) and by clinicians' rating through the HRSD-24 (24), acute suicidality assessed by the Beck Scale for Suicide Ideation (BSSI) (31), a history of previous suicidal attempts (yes/no), generalized and phobic anxiety measured by the Brief Symptom Inventory (BSI) (32) and the Generalized Anxiety Disorder Scale (GAD-7) (33), as well as comorbidity of any Axis I or II disorder diagnosed by the Structured Clinical Interview for *DSM-IV-TR* Axis I Disorders (SCID-I) (34) and the Structured Clinical Interview for *DSM-IV* Axis II Personality Disorders (SCID-II) (35). For examining comorbid anxiety as a predictor and moderator, we decided to only use the BSI and GAD-7 as self-report questionnaires for several reasons: First, they are continuous scales representing the current expression of anxiety, thereby providing more variance for the statistical analyses compared to diagnoses made by the SCID-I, which are of binary character, thus containing less variance. Second, we assessed all forms of anxiety disorders by the SCID-I (both lifetime and current diagnoses), and to test all these variables as predictors and moderators would needlessly increase the number of statistical tests.Third, we have less missing cases for the BSI and GAD-7 compared to the SCID-I.

#### Global, Psycho-Social Functioning, and Quality of Life

Baseline degree of global functioning and overall psychiatric burden assessed by the Global Assessment Functioning Scale (GAF) (36), dysfunctional social attitudes assessed by the Social Adaptation Self-Evaluation Scale (SASS) (37) and impairment of life quality through depression assessed by the Quality of Life in Depression Scale (QLDS) (38).

#### Interpersonal Problems

Self-reported, repeatedly occurring difficulties in interpersonal relationships assessed on the eight scales of the Inventory of Interpersonal Problems (IIP-64) (39); these are: domineering, suspicious/ distrustful, cold, socially inhibited, non-assertive, overly accommodating, self-sacrificing, and intrusive.

#### Childhood Trauma

Retrospective, self-reported forms of childhood trauma before the age of 18 assessed on the five scales of the Childhood Trauma Questionnaire (CTQ) (40). In this analysis, we defined the presence of the different types of childhood maltreatment as at least moderate-to-severe, corresponding to a pre-defined, specific cut-off of the respective scale set by Bernstein and Fink (41): emotional abuse (≥ 13 points), emotional neglect (≥ 15 points), physical abuse (≥ 10 points), physical neglect (≥ 10 points), and sexual abuse (≥ 8 points).

#### Treatment History

Previous underwent antidepressant medication received for a minimum of 4 weeks, psychotherapy underwent for at least eight sessions, a combination of both, as well as any form of previous inpatient treatment (yes/no).

#### Treatment Preference for Psychotherapy

All patients were asked to indicate which treatment option they generally prefer: antidepressant medication alone; psychotherapy alone; combined treatment of antidepressant medication and psychotherapy; or no preference. In the present analysis, we classified the answers in preferring psychotherapy (=1) or not (=0; all other options).

#### Treatment Expectancy

Self-ratings of the expected depression severity at week 48 assessed by the e-IDS-SR, which is an unpublished adaptation of the IDS-SR, used in this trial.

There is a large overlap with those baseline variables tested in the previous analysis relying on the combined moderator (19); however, due to an insufficient moderator effect size, not all tested baseline variables were entered as moderators into the final regression analysis there. In this analysis, we tested all enumerated variables as both individual predictor *and* moderator, enabling to discuss the roles of each one of these variables in conclusion.

### Treatment Outcome

The main outcome variable for all predictor/ moderator analyses was the HRSD-24 total score at week 48. Both groups did not differ in their baseline HRSD-24 scores (CBASP: mean=24.50, sd=7.60; SP: mean=25.18, sd=6.63; *p*=0.50).

### Statistical Analyses

All statistical analyses were performed on treatment completers, i.e patients who completed the whole therapy program of 32 sessions of CBASP or SP and presented valid HRSD-24 ratings at week 48. Between-group analyses were conducted to compare general differences in post-treatment scores (*Student's t*-test). We tested differences in demographic variables between patients allocated to CBASP and those allocated to SP, as well as between completers and non-completers (i.e., patients who dropped out from the trial before week 48).

With regard to the predictor and moderator analyses, linear regression models were built as depression severity was a continuous outcome. By following the recommendations of Kraemer et al. (42), we first z-standardized all continuous baseline variables in order to facilitate the interpretation of their effects. Predictors were defined as those baseline variables that showed a significant main effect in predicting the outcome without demonstrating an interaction with the treatment group variable, while moderators were defined as baseline variables that interacted with the treatment group variable in predicting the outcome, independently of the significance of the main effect (42). Models were built for each candidate baseline variable separately and were adjusted for study site and baseline depression severity, which were implemented as covariates into the models. Models testing predictors thus contained the main effects of study site, standardized baseline HRSD-24 scores, treatment group and the respective candidate baseline variable. For identifying moderators, separate models were built by adding the interaction term of the candidate variable and the treatment assignment to the main effects of the predictor model accordingly. Results are presented by regression coefficients and reported as significant at the conventional threshold of *p* < 0.05, two-sided. Analyses were performed with STATA 15.1 (Stata Corp, College Station, Texas).

## Results

From the *N* = 268 randomized outpatients, *N* = 209 completed the 48-week treatment program with 32 sessions of either CBASP (*N* = 113) or SP (*N* = 96). For a detailed description of the completer population, see Table 1. At baseline, the only significant difference between CBASP and SP completers was a higher percentage of employment in the group treated with CBASP. We found no significant differences in baseline variables between completers and non-completers (see Table 2 for descriptive statistics).

**Table 1:** Sociodemographic and clinical characteristics of the completers subdivided by treatment arm.

**Variable**	**CBASP**	**SP**
	**(*N* = 113)**	**(*N* = 96)**
Age at randomization, mean (SD), y	45.20 (11.98)	45.78 (11.98)
Female sex, No. (%)	81 (71.7)	57 (59.4)
Single, No. (%)	47 (41.6)	43 (44.8)
Married or cohabiting, No. (%)	45 (39.8)	40 (41.7)
Separated, divorced or widowed, No. (%)	21 (18.6)	13 (13.5)
High level of education, No. (%)	73 (64.6)	56 (58.3)
Employed, No. (%)^*^	90 (79.6)	59 (61.5)
Working hours per week, mean (SD), h	24.46 (16.51)	21.36 (20.13)
Presence of at least one physical illness, No. (%)	8 (7.3)	5 (5.4)
Subtype, No. (%)		
Double depression	47 (42.3)	43 (46.7)
Chronic MDD	35 (31.5)	31 (33.7)
Recurrent MDD with incomplete remission between episodes	29 (26.1)	18 (19.6)
Age at illness onset, mean (SD), y	13.01 (4.41)	13.02 (4.49)
Illness duration, mean (SD), y	32.19 (13.80)	32.77 (13.18)
HRSD-24 baseline score, mean (SD)	24.50 (7.60)	25.18 (6.63)
Remitters, No. (%)	41 (36.3)	24 (25.0)

HRSD-24, 24-Item Hamilton Rating Scale for Depression; MDD, major depressive disorder; SD, standard deviation; y, years.

* *Significant between-group difference at p = 0.004*.

**Table 2:** Differences in baseline variables between completers and non-completers.

**Baseline variable**	**Completers**	**Non-completers**	
**Continous variables**	**Mean (SD)**	**Mean (SD)**	***p***
Age at randomization	45.47 (11.96)	42.93 (11.18)	0.15
Age at illness onset	13.01 (4.44)	12.95 (4.36)	0.92
Illness duration (y)	32.45 (13.49)	29.98 (12.51)	0.21
IDS-SR score	38.90 (9.82)	38.83 (8.33)	0.96
HRSD-24 score	24.81 (7.16)	24.70 (6.41)	0.91
BSSI score	6.30 (7.19)	7.49 (7.95)	0.30
GAD-7 score	10.86 (4.65)	11.02 (4.20)	0.83
BSI anxiety score	6.14 (3.78)	6.58 (3.82)	0.45
BSI phobia score	2.62 (2.48)	3.17 (2.76)	0.16
GAF score	54.38 (9.25)	54.09 (8.87)	0.84
SASS score	30.22 (6.55)	29.39 (6.19)	0.41
QLDS score	18.91 (7.70)	19.98 (7.72)	0.37
IIP-64 total score	14.89 (3.63)	14.77 (3.83)	0.83
**Binary variables**	**N**	**N**	***p***
Female gender	138	39	0.99
Single	90	27	0.71
Married or cohabiting	85	21	0.48
Separated, divorced or widowed	34	11	0.67
High level of education	129	43	0.11
Employed	149	41	0.79
Presence of morbidities (≥1 physical illness)	13	2	0.37
Chronic MDD	66	16	0.52
Double depression	90	29	0.38
Recurrent MDD with incomplete remission between episodes	47	12	0.74
History of suicidal attempts	58	18	0.47
Any Axis I disorder^a^	87	26	0.74
Any Axis II disorder^a^	82	21	0.61
Early physical abuse^b^	42	13	0.55
Early physical neglect^b^	61	21	0.18
Early emotional abuse^b^	119	32	0.82
Early sexual abuse^b^	48	9	0.99
Early emotional neglect^b^	132	35	0.76
Prior medication^c^	117	31	0.64
Prior psychotherapy^d^	117	36	0.49
Prior combination therapy^e^	39	14	0.39
Prior inpatient treatment^f^	105	33	0.44
Preference for psychotherapy	157	41	0.47

BSI, Brief Symptom Inventory; BSSI, Beck Scale for Suicidal Ideation; CTQ, Childhood Trauma Questionnaire; GAD-7, Generalized Anxiety Disorder Scale-7; GAF, Global Assessment Functioning Scale; HRSD-24, 24-Item Hamilton Rating Scale for Depression; IDS-SR, self-rated Inventory of Depressive Symptomatology; IIP-64, Inventory of Interpersonal Problems; MDD, major depressive disorder; QLDS, Quality of Life in Depression Scale; SASS, Social Adaptation Self-Evaluation Scale; y, years.

a Diagnosed by the SKID-I or SKID-II according to DSM-IV classification.

b Presence indicates a clinical severity of at least moderate to severe on the CTQ.

c History of ≥ 4 weeks of treatment with antidepressant medication.

d History of ≥ 8 sessions of psychotherapy.

e History of combination treatment with antidepressant medication (≥ 4 weeks) and psychotherapy (≥ 8 sessions).

f *History of any kind of psychiatric inpatient treatment*.

The between-group comparisons at week 48 revealed that CBASP completers had significantly lower HRSD-24 scores (CBASP: mean = 13.96, sd = 9.56; SP: mean = 16.69, sd = 9.87; *p* = 0.044).

### Predictors of Depression Severity at Week 48

In total, our analyses identified 10 predictors (all main effects with *p* < 0.05): Higher HRSD-24 scores at week 48 were predicted by higher baseline scores on the HRSD-24 scale, BSSI scale, BSI anxiety scale, GAD-7 scale, and IIP-64 social inhibition scale. In addition, higher HRSD-24 scores at week 48 were also predicted by the presence of early emotional or sexual abuse at baseline, as well as by the presence of at least one previous inpatient treatment. In contrast, lower HRSD-24 scores at week 48 were predicted by higher baseline scores on the SASS scale, as well as by the presence of preference for psychotherapy rated as baseline (for more details, please see Table 3).

**Table 3:** Predictors and moderators of depression severity at week 48.

	**Variable main effect**		**Variable x Group**		**Role**
**Baseline variable**	**B (95% CI)**	***p***	**B (95% CI)**	***p***	
**SOCIO-DEMOGRAPHY**					
Female gender^a^	0.50 (−2.14; 3.15)	0.71	−1.05 (−6.34; 4.23)	0.69	
Age at randomization^b^	0.72 (−0.53; 1.98)	0.26	−2.03 (−4.45; 0.39)	0.10	
Single^a^	0.44 (−2.11; 3.00)	0.73	1.91 (−3.12; 6.95)	0.45	
Married or cohabiting^a^	−1.07 (−3.61; 1.47)	0.41	0.97 (−4.17; 6.11)	0.71	
Separated, divorced or widowed^a^	1.11 (−2.28; 4.51)	0.52	−5.61 (−2.42; 1.20)	0.17	
High level of education^a^	−0.11 (−2.70; 2.48)	0.93	−0.83 (−5.94; 4.29)	0.75	
Employed^a^	−1.65 (−4.47; 1.17)	0.25	2.68 (−2.90; 8.27)	0.34	
Working hours per week^b^	−0.27 (−1.64; 1.09)	0.69	−0.75 (−3.57; 2.07)	0.60	
Presence of morbidities (≥1 physical illness)^a^	1.53 (−3.57; 6.63)	0.55	−2.50 (−12.97; 7.97)	0.64	
**CLINICAL CHARACTERISTICS**					
Double depression^a^	0.11 (−2.53; 2.76)	0.93	−1.09 (−6.25; 4.07)	0.68	
Chronic MDD^a^	0.94 (−1.92; 3.81)	0.52	−3.51 (−8.98; 2.96)	0.21	
Recurrent MDD with incomplete remission between episodes^a^	−1.32 (−4.49; 1.84)	0.41	6.18 (0.16; 12.20)	0.044^*^	M
Age at illness onset^b^	0.36 (−0.91; 1.63)	0.57	1.55 (−0.92; 4.03)	0.22	
Illness duration^b^	0.53 (−0.73; 1.79)	0.41	−2.39 (−4.82; 0.04)	0.054	
HRSD-24 score^b^	2.43 (1.17; 3.70)	<0.001^*^	−1.10 (−3.62; 1.41)	0.39	P
IDS-SR score^b^	1.50 (−0.11; 3.11)	0.069	−3.68 (−6.14; −1.21)	0.004^*^	M
BSSI score^b^	2.32 (0.93; 3.71)	0.001^*^	1.13 (−1.45; 3.72)	0.39	P
History of suicidal attempts^a^	0.28 (−2.58; 3.14)	0.85	−4.33 (−10.00; 1.33)	0.13	
BSI anxiety score^b^	1.80 (0.38; 3.23)	0.014^*^	−1.83 (−4.31; 0.66)	0.15	P
BSI phobia score^b^	1.10 (−0.36; 2.56)	0.14	−0.35 (−2.96; 2.27)	0.79	
GAD-7 score^b^	1.57 (0.14; 2.99)	0.031^*^	−2.13 (−4.59; 0.32)	0.09	P
Any Axis I disorder^a, c^	1.43 (−1.21; 4.08)	0.29	−6.02 (−11.04; −0.99)	0.019^*^	M
Any Axis II disorder^a, c^	2.25 (−0.51; 5.01)	0.11	0.03 (−5.14; 5.21)	0.99	
**FUNCTIONALITY AND QUALITY OF LIFE**^**b**^					
GAF score	0.25 (−1.49; 1.99)	0.78	2.12 (−0.45; 4.70)	0.11	
SASS score	−2.05 (−3.39; −0.72)	0.003^*^	1.06 (−1.42; 3.54)	0.40	P
QLDS score	0.85 (−0.63; 2.33)	0.26	−1.19 (−3.80; 1.41)	0.37	
**INTERPERSONAL PROBLEMS**^**b, d**^					
Domineering	−0.46 (−1.80; 0.88)	0.50	−2.33 (−4.93; 0.28)	0.08	
Suspicious/distrustful	0.92 (−0.42; 2.26)	0.18	−1.31 (−3.99; 1.38)	0.34	
Cold	1.06 (−0.24; 2.37)	0.11	−1.32 (−3.91; 1.27)	0.32	
Socially inhibited	2.34 (1.04; 3.65)	0.001^*^	−1.35 (−3.86; 1.15)	0.29	P
Non-assertive	1.04 (−0.29; 2.38)	0.13	−1.14 (−3.70; 1.41)	0.38	
Overly accommodating	1.00 (−0.33; 2.33)	0.14	−1.45 (−3.95; 1.06)	0.26	
Self-sacrificing	0.76 (−0.56; 2.07)	0.26	−2.02 (−4.52; 0.48)	0.11	
Intrusive	−0.15 (−1.43; 1.13)	0.82	−0.67 (−3.24; 1.89)	0.60	
**EARLY TRAUMA**^**a, e**^					
Emotional abuse	3.40 (0.79; 6.01)	0.011^*^	−3.93 (−9.00; 1.14)	0.13	P
Emotional neglect	2.81 (0.08; 5.53)	0.043^*^	−6.72 (−12.04; −1.41)	0.013^*^	M
Physical abuse	−0.91 (−4.14; 2.33)	0.58	−4.09 (−10.39; 2.20)	0.20	
Physical neglect	1.44 (−1.37; 4.26)	0.31	−7.06 (−12.51; −1.61)	0.011^*^	M
Sexual abuse	6.03 (3.17; 8.88)	<0.001^*^	0.81 (−4.89; 6.52)	0.78	P
**PREVIOUS TREATMENTS**^**a**^					
Medication^f^	1.27 (−1.33; 3.87)	0.34	−5.58 (−10.50; −0.65)	0.027^*^	M
Psychotherapy^g^	1.80 (−0.71; 4.30)	0.16	0.95 (−4.11; 6.02)	0.71	
Combination^h^	1.94 (−1.26; 5.14)	0.23	−2.79 (−9.14; 3.55)	0.39	
Inpatient^i^	4.52 (2.00; 7.04)	0.001^*^	−4.41 (−9.24; 0.40)	0.07	P
***Preference for psychotherapy**^**a**^*	−3.01 (−6.00; −0.01)	0.049^*^	−2.64 (−8.56; 3.28)	0.38	P
***Therapy expectancy**^**b**^*	0.64 (−0.60; 1.88)	0.31	−2.08 (−4.53; 0.36)	0.09	

BSI, Brief Symptom Inventory; BSSI, Beck Scale for Suicidal Ideation; CI, confidence interval; CTQ, Childhood Trauma Questionnaire; GAD-7, Generalized Anxiety Disorder Scale-7; GAF, Global Assessment Functioning Scale; HRSD-24, 24-Item Hamilton Rating Scale for Depression; IDS-SR, self-rated Inventory of Depressive Symptomatology; IIP-64, Inventory of Interpersonal Problems; M, moderator; MDD, major depressive disorder; P, predictor; QLDS, Quality of Life in Depression Scale; SASS, Social Adaptation Self-Evaluation Scale.

a Categorical variable (0=no; 1=yes).

b Z-standardized continuous variable (0=mean; 1=mean + 1SD).

c Diagnosed by the SKID-I or SKID-II according to DSM-IV classification.

d As assessed by the IIP-64.

e Presence indicates a clinical severity of at least moderate to severe on the CTQ.

f History of ≥ 4 weeks of treatment with antidepressant medication.

g History of ≥ 8 sessions of psychotherapy.

h History of combination treatment with antidepressant medication (≥ 4 weeks) and psychotherapy (≥ 8 sessions).

i History of any kind of psychiatric inpatient treatment.

* *significant at p < 0.05*.

### Moderators of Depression Severity at Week 48

**Figure 1:**
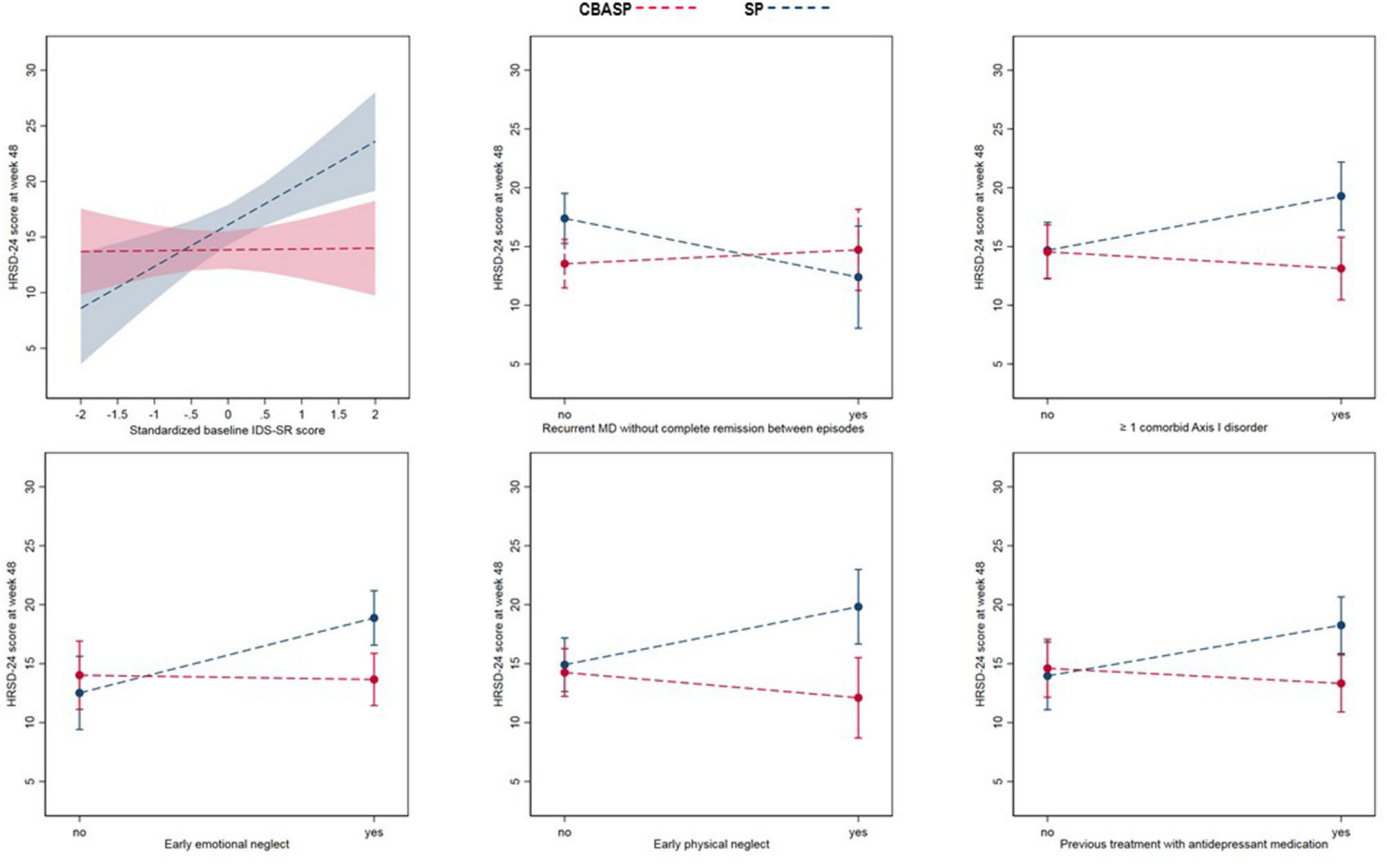
Moderators of depression severity at week 48. IDS, self-rated Inventory of Depressive Symptomatology; HRSD-24, 24-Item Hamilton Rating Scale for Depression; MDD, major depressive disorder.

Baseline variables identified as moderators of lower post-treatment HRSD-24 scores for patients treated with CBASP were: Higher levels of self-rated depression severity (IDS-SR scores), comorbidity of at least one Axis I disorder, a history of childhood moderate-to-severe emotional or physical neglect (CTQ scales), and a history of at least one previous treatment with antidepressant medication. This means that CBASP patients showing these features at baseline had lower post-treatment scores at week 48 than those with similar features treated with SP. Concerning the PDD subtype, we found a crossover-effect in that patients with chronic MDD and Double Depression treated with CBASP had lower post-treatment scores at week 48 than those with these features treated with SP. In line with this, those classified to have a recurrent MDE without complete remission between the episodes benefited more from SP than from CBASP (Table 3). Figure 1 illustrates all six identified moderators by plots of their interaction effects with the treatment group. All other baseline variables lacked statistical significance for being declared as predictors or moderators (all *p* > 0.05).

## Discussion

In a large randomized clinical trial conducted in adult, antidepressant-free outpatients with early-onset PDD, CBASP has been shown to outperform SP with response rates of 38,7% compared to 24,3% at the end of the extended treatment phase after 48 weeks (7). In this secondary-analysis conducted in patients who completed the interventions of this randomized clinical trial, we examined the roles of a wide range of baseline variables as predictors and moderators of the effectiveness of CBASP and SP on depression severity at the end of the extended treatment phase at week 48.

In terms of predictors, we found that a poor response to both psychotherapies was predicted by a higher baseline severity of depression (higher HRSD-24 baseline scores), more pronounced suicidality (higher BSSI baseline scores), more intense anxiety (higher BSI anxiety and GAD-7 baseline scores), stronger social inhibition (higher IIP-64 baseline scores), a self-reported history of moderate-to-severe emotional or sexual abuse, as well as at least one inpatient treatment. Patients who had higher baseline levels of social functioning (higher SASS baseline scores) and a preference for psychotherapy had, contrarily, lower levels of depression severity at week 48 independent of the assigned treatment form.

The findings of the performed predictor analyses largely confirmed our hypotheses and are in line with previous research confirming that those patients who were initially more mentally stable (i.e less depressed, less anxious, less suicidal), higher socially functioning and preferring psychotherapy, responded better to both treatments when compared to patients on the other side of the respective continuum or category. It is reasonable that a less pathological and higher functioning baseline status has facilitated the psychotherapeutic learning and enabled a better recovery process in both groups. Moreover, the confirmed positive impact of having a preference for psychotherapy on the outcomes of both psychotherapies is in line with previous results (21, 22) and supports the conclusion that psychotherapy is more effective and recommendable than other treatments options for PDD patients who prefer psychotherapy over other alternative treatments for depression (9).

From the opposite perspective, we can also conclude that patients who were initially more pathologic benefitted less from both therapies. Thus, for more severely affected patients, both psychotherapies might be insufficient for achieving significant symptom reductions when delivered as monotherapies, as was the case in this trial. These subpopulations might respond better to a combined approach between antidepressant medication and person-centered psychotherapy which flexibly and adaptively combines unspecific, transdiagnostic, and disorder-specific interventions. For example, it has been shown that the combination of CBASP and an antidepressant medication was more effective for PDD patients with a higher baseline symptom severity and pronounced anxiety (43, 44) than monotherapy with CBASP, indicating that an augmentation with pharmacotherapy is more recommendable for these patients than treatment with CBASP alone (7). This conclusion has also been supported in a participant data network meta-analysis which compared the effectiveness of CBASP as monotherapy to that of antidepressant medication and their combination (20). In a 2-year follow-up study of this trial, Schramm et al. (45) evaluated the effects of CBASP and SP one and two years after treatment termination. CBASP outperformed SP in the number of well weeks with no/minimal symptoms, self-rated depressive symptoms, and depression-related quality of life one year after treatment termination, but not after two years. This result could be strongly attributed to a worsening of symptoms in the subgroups marked by baseline characteristics here identified as predictors, who benefitted less favorable from both interventions, and indicates the necessity of maintenance treatment for PDD patients.

Interestingly, we detected a lower effectiveness of both interventions for patients reporting a history of moderate-to-severe early emotional or sexual abuse, while CBASP was found to be more effective than SP for patients reporting early emotional or physical neglect. These results suggest that early-life trauma in form of abuse might be an important factor that contributes to non-response to psychotherapy in chronically depressed patients, while cognitive-behavioral consequences of early neglect might be modifiable by disorder-specific psychotherapy with CBASP. If different types of early trauma are associated with different responses to psychotherapy, then this information may prove crucial in designing and selecting optimal treatments for chronically depressed patients.

Finally, treatment expectancy had no influence on the post-treatment depression severity in our trial. We did not identify predictors or moderators from the socio-demographic domain, which could be attributable to the relatively homogeneous population of this trial (7).

In terms of moderators, CBASP displayed a multifaceted superiority over SP, meaning that patients with an elevated self-perceived depression severity (higher IDS-SR baseline scores), no recurrent MDE without complete remission between the episodes, comorbidity of Axis-I disorders, a history of at least one previous antidepressant treatment, and, as mentioned before, early trauma in form of moderate-to-severe emotional or physical neglect, had a lower depression severity at week 48 when treated with CBASP than those who were treated with SP. These results are in line with the previous moderator analysis (19) based on the data of this trial, which applied a modern machine learning method in order to identify subgroups of patients who respond better to CBASP than to SP and vice versa. With except of previous antidepressant medication, all here identified moderators had a moderator effect size large enough to be entered into the final regression model used in the analysis by Serbanescu et al. (19) to combine the most relevant moderators in order to exploratory identify the subgroups. The fact that the moderating role of these variables could be replicated in this more classical analysis underlines its robustness and validity in this trial. A more detailed interpretation of the moderating role of these variables is provided in the previous article (19). As emphasized there, these promising findings are in need of additional detailed investigations in order to be understood, as well as replication in future trials for enabling reliable treatment choice recommendations for the clinical practice.

This study has a number of important strengths: First, the antidepressant-free status of the patients allows ascribing the findings to the two tested psychotherapies alone. Second, we tested a relatively wide range of baseline characteristics. Third, the here performed analysis provides evidence for predictors as well as for moderators of two widely used therapies. We tested a relatively high number of variables, yielding many interesting results that open new questions which remain to be further investigated. However, some limitations must be also considered: Possible undesired, side-effects including transient worsening of symptoms and transient risk of suicidality at the beginning of therapy or in the context of unexpected psychosocial stress might have occurred in both treatment groups, and were not subject of this analysis. As a further limitation, our sample included only medication-free patients who were evaluated as enough mentally stable to be able to participate in the study. It can be assumed that the effectiveness of both therapies would have been smaller in more severely depressed patients. The exclusion criteria of the trial therefore may limit the generalizability of the findings to the general PDD population. Furthermore, the therapy duration of 48 weeks has revealed numerous clinically relevant predictors and moderators, but may be very resource-intensive for implementation in clinical practice. Finally, given the exploratory approach and large number of performed tests, the possibility of false positive findings has to be taken into account when considering the results. Thereby, our results need replication in future trials in order to permit valid treatment choice recommendations.

## Conclusion

A multifactorial combination between elevated depression severity, suicidality, traumatic childhood experiences characterized by abuse, social inhibition and anxiety may represent the basis of non-response to psychotherapy in patients with PDD and consequently contribute to the persistence of the illness and its refractoriness. Nevertheless, disorder-specific psychotherapy with CBASP might be more effective and recommendable for a variety of particularly burdened patients with PDD than Supportive Psychotherapy. Further personalized clinical research is needed in order to understand and develop the (combination of) treatments that meet the needs of the most affected patients with PDD.

## Data Availability Statement

The data analyzed in this study is subject to the following licenses/restrictions: The dataset of the initial clinical randomized trial is not available to the public. Requests to access these datasets should be directed to elisabeth.schramm@uniklinik-freiburg.de.

## Ethics Statement

The studies involving human participants were reviewed and approved by Ethics Committee of the University of Freiburg, University of Bonn, University of Heidelberg, University of Tübingen, University Medical Center Hamburg-Eppendorf, University of Marburg, and University of Lübeck. The patients/participants provided their written informed consent to participate in this study.

## Author Contributions

IS and DS: had full access to all of the data in the study and take responsibility for the integrity of the data and the accuracy of the data analysis and drafting of the manuscript. IS: was responsible for statistical data analysis. IS, HW, SD, and DS: study concept and design. IS, HW, SD, JK, IZ, MB, MHa, RM, MHä, ES, and DS: acquisition, analysis, or interpretation of data. IS, BW, IZ, RM, MHa, MHä, DS, and ES: administrative, technical, or material support. All authors gave critical revision of the manuscript for important intellectual content.

## Conflict of Interest

ES received modest book royalties and honoraria for workshops and presentations relating to CBASP. DS received honoraria for several workshops and presentations relating to CBASP. JK received payments for workshops and books (Beltz, Elsevier and Hogrefe) on psychotherapy for chronic depression. MB received honoraria for workshops and presentations relating to CBASP. The remaining authors declare that the research was conducted in the absence of any commercial or financial relationships that could be construed as a potential conflict of interest.
